# MEN WITH PARKINSON'S MAY HAVE GREATER DISEASE BURDEN IN ASPECTS OF COGNITIVE AND PSYCHOSOCIAL FUNCTION THAN WOMEN

**DOI:** 10.1093/geroni/igz038.3445

**Published:** 2019-11-08

**Authors:** Madeleine E Hackney, Eeshani Singh, Ella Leeth, Allison Bay, Liang Ni

**Affiliations:** 1 Department of Medicine, Emory University School of Medicine, Atlanta, Georgia, United States; 2 Emory University College of Arts and Sciences, Atlanta, Georgia, United States; 3 Emory University School of Medicine, Atlanta, Georgia, United States

## Abstract

Early in PD, women may experience a more benign disease course than man. Limited research has examined differences between men and women with moderate, treated PD with a mean duration >5 y. Retrospective analyses were performed on data collected from studies, conducted 2011–2019, that assessed motor, cognitive and psychosocial function in 199 people with PD (women=72). We compared performance using univariate analyses, adjusting for age, housing type and education. Men and women patients were not different in PD stage (Stage Mdn= 2, IQR=.5), age (mean±SD; 69.1± 8.9 y), education (16.4 ± 2.3 y), number of medications (5.9±4.1), comorbidities (3.4±1.8), physical function, nor time with PD (6.6±4.6 y). Women were more likely to live in assisted living or senior residences (p=.005). Men gave more correct answers in subtraction, (8.8 ± 4.0 vs. 6.4±3.7; p<.001) but sexes did not differ in percent correct. On the MDS-UPDRS, Men exhibited more burden in subjectively rated non-motor (13.4 ± 7.6 vs. 10.7±7.3; p=.013), and motor experiences of daily living (16.9±8.9 vs. 10.6±7.1; p<.001) and motor symptoms (34.1 ± 12.1 vs. 31.8±12.2; p=.014). Men performed worse at inhibition (6.4±4.6 vs. 7.8 ± 5.0; p=.014) but made fewer errors on inhibition/switching (7.0±3.9 vs.7.8±4.4; p=.05). Men had higher depression scores: 12.5±8.9 vs. 9.4±7.8; p=.016. No differences in performance on spatial cognition were noted. Men with moderate PD were more depressed, had worse motor and cognitive function, non-motor and motor experiences of daily living and motor symptoms than women. Sex-tailored therapies may reduce differences in performance between sexes.

